# Synergetic Anion–Cation Redox Ensures a Highly Stable Layered Cathode for Sodium‐Ion Batteries

**DOI:** 10.1002/advs.202105280

**Published:** 2022-04-07

**Authors:** Xiang Li, Jialiang Xu, Haoyu Li, Hong Zhu, Shaohua Guo, Haoshen Zhou

**Affiliations:** ^1^ Center of Energy Storage Materials & Technology College of Engineering and Applied Sciences Jiangsu Key Laboratory of Artificial Functional Materials National Laboratory of Solid State Microstructures and Collaborative Innovation Center of Advanced Microstructure Nanjing University Nanjing 210093 China; ^2^ College of Chemistry Zhengzhou University Zhengzhou 450001 China; ^3^ University of Michigan‐Shanghai Jiao Tong University Joint Institute Shanghai Jiao Tong University Shanghai 200240 China; ^4^ Shenzhen Research Institute of Nanjing University Shenzhen 51800 China

**Keywords:** anion redox, high‐stable layered cathodes, sodium‐ion batteries, suppressed phase transition

## Abstract

Sodium‐ion batteries are commonly regarded as a promising candidate in large‐scale energy storage. Layered iron/manganese oxide cathodes receive extensive attentions due to the element abundance and large theoretical capacity. However, these materials usually undergo obvious degradation of electrochemical performance due to the tendency of Mn dissolution and Fe migration during continuous sodium release and uptake. Herein, a strategy of anion–cation synergetic redox is proposed to suppress the structural deterioration originated from overusing the electrochemical activity of transition‐metal ions, and decreased lattice strain as well as superior electrochemical performance are realized simultaneously. Results show that the Na_0.8_Li_0.2_Fe_0.2_Mn_0.6_O_2_ (NLFM) electrode is highly resistant to the erosion of moisture that is distinct from the traditional Mn/Fe‐based electrodes. Moreover, the NLFM electrode demonstrates solid solution behavior without phase transition during cycles. The ultra‐small volume change of 0.85% is ascribed to the negligible manganese dissolution and invisible transition‐metal migration. The high‐stable layered structure assures superior reversible capacity of ≈165 mA h g^–1^, excellent rate capability, and splendid capacity retention of over 98.3% with 100 cycles. The findings deepen the understanding of the synergy between anion and cation redox and provide new insights to design the high‐stable layered cathode for sodium‐ion batteries.

## Graphical abstract

A strategy of anion–cation synergetic redox is proposed to suppress the structural deterioration of sodium‐ion layered Fe/Mn‐based cathodes. The electrode is of high resistance to the erosion of moisture that is distinct from the traditional electrodes. Moreover, the electrode demonstrates solid solution behavior without phase transition during cycles. The ultra‐small volume change of 0.85% is ascribed to the negligible manganese dissolution and invisible Fe migration. The high‐stable layered structure assures superior reversible capacity and excellent rate capability.

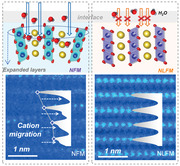

## 1 Introduction

Sodium‐ion batteries (SIBs) have caused extensive concern due to the promising application in large‐scale energy storage over recent years.^[^ ^1^ ^]^ Layered oxide cathodes have been mostly studied as a cathode for SIBs, and manganese/iron‐based ones based on the abundant materials and high theoretical capacity are increasingly attracting the attention.^[^ ^2^, ^3^, ^4^, ^5^, ^6^, ^7^, ^8^, ^9^ ^]^ For the large‐scale energy storage devices, their cost‐effective characteristic ensures the layered Mn/Fe‐based oxides potentially competitive with LiCoO_2_, namely the system most widely used in Li‐ion batteries. However, some key issues remain to be solved:^[^ ^10^, ^11^, ^12^, ^13^, ^14^ ^]^ 1) The Mn‐based layered oxides usually suffer from the manganese dissolution due to overexploiting the manganese activity; 2) Fe‐ion migration from TM (transition‐metal) slabs to Na layers are inclined to occur in Fe‐containing layered oxides, blocking the Na‐ion continuous (de)intercalation;^[^ ^8^, ^15^ ^]^ 3) These Mn/Fe layered oxides are unstable when exposed to the moisture air, increasing the cost of material storage and transportation. In addition, the irreversible phase transitions also restrict the electrochemical performance of the materials.^[^ ^12^, ^13^, ^14^ ^]^

Herein, we propose a strategy of synergetic anion–cation redox via Li substitution in layered Mn/Fe‐based cathodes, Na_2/3_Fe_2/3_Mn_1/3_O_2_ (hereafter denoted as NFM), to overcome the above difficulties. It is shown that the obtained Na_0.8_Li_0.2_Fe_0.2_Mn_0.6_O_2_ (hereafter denoted as NLFM) shows at least three improvements owing to Li substitution. First, NLFM displays impressive structural stability after exposing in the air for 1 month, and even soaking in water for 1 week. While a new phase appears obviously in NFM after storing in both air and water. Besides, oxygen redox process is activated to offer improved specific capacity, as evidenced by the results of density functional theory (DFT) calculations, soft X‐ray absorption spectroscopy (sXAS), and X‐ray photo‐electron spectroscopy (XPS). Furthermore, the detrimental phase transition of NFM is dramatically suppressed in NLFM, which contributes to fast Na^+^ transporting and better cycling performance. The negligible Mn dissolution, invisible Fe migration, and lattice strain are confirmed as well. As a result, NLFM demonstrates an enhanced electrochemical performance, represented by high reversible capacity of ≈165 mA h g^–1^, improved capacity retention (98.3% after 100 cycles at 200 mA g^–1^), and robust capacity restorability. These superiorities promote NLFM a promising electrode for sodium‐ion batteries applied in sustainable and large‐scale energy storage.

In NLFM, Li substitution in the transition‐metal slabs generates the possibility of anionic redox, similar to the typical Na‐rich, Li‐rich, and analogous materials.^[^ ^16^, ^17^, ^18^, ^19^ ^]^ It is noted that anionic redox behavior is generally considered as a handicap for structural stability, aggravating phase transition, and energy deficiency. However, in our research, anionic redox plays a leading role in the structural stability of NLFM. The charge compensation of oxygen prevents Mn from overexploiting, which abates severe Mn dissolution. Besides, the interaction between the oxidized oxygen protects the crystal structure from phase transition, resulting in the negligible volume change of 0.85% during cycling. Briefly, the synergy between anion and cation redox is pivoted in NLFM. We hope our results could offer a better understanding of this synergy effect and provide new considerations for designing cathodes with high energy density as well as stability.

**Figure 1:**
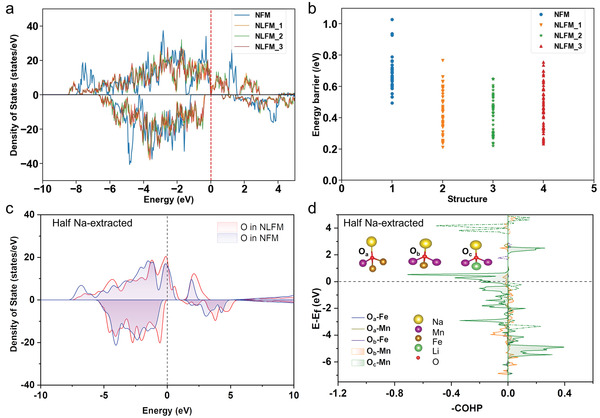
a) DOS of oxygen atoms in pristine NFM and different structures of NLFM. b) Energy barriers of Na migration in NFM and NLFM. Computed diffusion paths and energy profiles using CAVD+BVSE. c) Total DOS of oxygen atoms in NLFM‐half and NFM‐half. d) Bonding information of O‐TM for pristine and after extraction of half sodium ions. ‐COHP <0, =0, and >0 represent antibonding, nonbonding, and bonding, respectively. Line and dotted line represent the up spin and down spin, respectively. O‐TM (Fe, Mn) means the bonding information between oxygen atoms with different transition metals.

DFT calculations were first considered to better understand the anionic redox process in NLFM. The density of states (DOS) calculations of NFM were performed based on DFT, as shown in **Figure** **1**a. (See details of the methods and the structure models in Figures S1−S3, Supporting Information). The peak of DOS in NLFM appears above the Fermi energy, which is different from NFM. NFM presents semiconductor‐like properties and NLFM presents metallic‐semiconductor properties. NLFM structures are complex and the path of Na migration has many possibilities. The energy barriers of all the Na migration paths in NFM and NLFM (with three preferential structures named NLFM_1, NLFM_2, NLFM_3) were calculated in the CAVD (crystal structure analysis by Voronoi decomposition)+BVSE (bond valence site energy) method,^[^ ^20^ ^]^ as shown in Figure 1b. The energy barrier of NFM is 0.494–1.027 eV, which is larger than 0.210–0.766 eV of NLFM. Thus, NLFM is inferred to have a larger Na diffusion than NFM.

The DOS calculations of oxygen atoms and the crystal orbital Hamilton population (COHP) analyses for both half Na‐extract NFM (denoted as NFM‐half) and NLFM‐half were performed based on DFT, as shown in Figure 1c,d. Note that all the local atomic environment of oxygen atoms is the same in NFM‐half, which are surrounded by Na, Fe, and Mn atoms (denoted as O_a_). In contrast, there are two different configurations for oxygen atoms in NLFM‐half, denoted as O_b_ (surrounded by Na, Fe, and Mn atoms with a different Fe/Mn ratio compared to O_a_) and O_c_ (surrounded by Na, Li, and Mn atoms). The total DOS of the oxygen atoms are shown in Figure 1a. The DOS of the oxygen atoms surrounded by Na, Fe, and Mn, in NFM‐half are different from that of O in NLFM especially between 1 and −1 eV, where Li ions are near the oxygen atom.

It has been reported before that an isolated O 2p orbital is present along the direction where oxygen is linearly bonded with two Li atoms (Li—O—Li configuration).^[^ ^21^ ^]^ In NLFM‐half, O_c_ atoms are in a similar environment (Na—O—Li configuration). This result indicates that the labile electrons from the O ion in NLFM‐half likely originate from this particular Na—O—Li configuration and provide possibilities for oxygen to participate the anionic redox for charge compensation.

COHP shows bonding/antibonding states as an efficient and reliable tool to extract the chemical‐bonding information. Figure 1d shows that both positive and negative COHP of O‐TM (transition metal) exists in NFM‐half and NLFM‐half. The bonding and antibonding interaction of O_c_‐Mn are much stronger than O_a_‐Mn and O_b_‐Mn, indicating an enhanced O‐Mn interaction with Li doping. Thus, the O near Li can be easier to participate in the anionic redox process. Moreover, **Table** **1** shows the minimum distance between adjacent oxygen atoms in NFM‐half and NLFM‐half.

**Table 1:** The minimum distance between adjacent oxygen atoms (Å)

Materials	Distance [Å]
Na_6_Fe_12_Mn_6_O_36_	2.6132
Na_6_Li_3_Fe_3_Mn_12_O_36_	2.4952

The local structure changes of NFM‐half, NLFM‐half, and the surrounding environment of O atom upon charging are displayed in Figure S4 (Supporting Information). O_a_ in NFM‐half and O_b_ in NLFM‐half are both only bonded to Fe, Mn, and Na atoms, while O_c_ in NLFM is also bonded to Li atom in addition to Mn and Na atoms. The COHP analysis (Figure 1d) indicates that both the antibonding and bonding strength under the Fermi level between Mn and O_c_ are larger than those between Mn and Oa/Ob, which is consistent with the shorter distance between Mn and O_c_. After doping with Li atoms, the local environment of O atoms is changed. In NLFM‐half, the Mn‐O_c_ bond length (≈1.885 Å) is shorter than Mn‐O_a_ (≈1.945 Å) in NFM‐half. But the Mn‐O_b_ bond length (≈2.010 Å) is longer than Mn‐O_a_ (≈1.945 Å) in NFM‐half. Compared with the transition metal in NFM, Li ions in NLFM can provide fewer electrons. When Li atoms replace transition metal atoms, more electrons in Mn atoms likely are activated and the Bader charge of Mn in NFLM‐half is smaller than NFM‐half. As shown in Table 1, it is obvious that the distance between adjacent oxygen atoms decreases after Li substitution, consistent well with the result of COHP.^[^ ^22^ ^]^

Furthermore, the Bader Charges (Figure S4, Supporting Information) represent the charge transfer of each atom. The charge of O_c_ in NLFM‐half is –0.985e, less negative than that of O_a_ in NFM‐half and O_b_ in NLFM‐half as shown in **Table** **2**, which may be attributed to the Na—O—Li configuration in NLFM‐half. A similar effect has been reported for Li‐rich material with Li—O—Li configuration, which changes the charge of oxygen atoms and gives rise to the anionic redox process.^[^ ^17^, ^23^ ^]^ During the extraction of sodium ions, the transition metal has more positive charges compared with Li, therefore the Bader charge of O_a_ is more negative than that of O_b_ and O_c_ after extraction (the charges of O_b_ and O_c_ are close to each other). Based on these findings, oxygen atoms will be easily oxidized after Li substitution.

**Table 2:** The Bader Charge of different elements. Mn_Li_ represents the Mn atom connected to Li atom and Mn represents the Mn atom without connecting with Li atom

	Bader Charge		
Samples	Mn	Fe	O_a_
Na_6_Fe_12_Mn_6_O_36_	1.868	1.809	−1.059

	Bader Charge					
Samples	Li	Mn_Li_	Mn	Fe	O_c_	O_b_
Na_6_Li_3_Fe_3_Mn_12_O_36_	0.884	1.861	1.861	1.840	−0.985	−0.971

**Figure 2:**
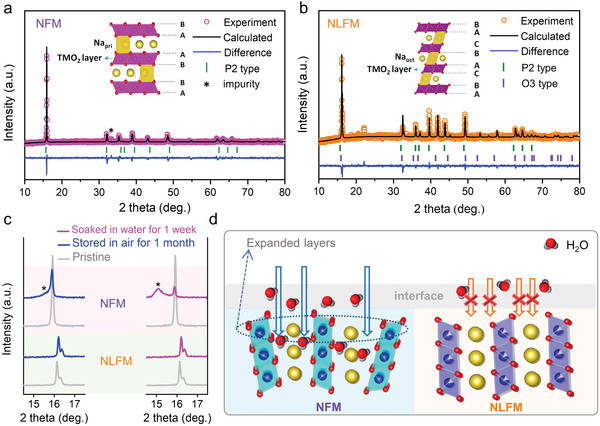
Structure characterization of NFM and NLFM. a) XRD patterns of NFM with Le Bail refinements. b) XRD patterns of NLFM with Le Bail refinements. Diagram of crystal P2 type structure and O3 type structure are inserted in (a) and (b). c) Comparison of XRD patterns for NFM and NLFM after exposing in air for 1 month and soaking in water for 1 week. d) Schematic diagram of two materials against CO_2_ and H_2_O.

The XRD patterns of NFM and NLFM are displayed in **Figure** **2**. Le Bail fits for calculating the crystal structures were employed by utilizing General Structure Analysis System/Experiment Graphical User Interface (GSAS/EXPGUI).^[^ ^24^, ^25^ ^]^ The diagrams of the crystal P2 type and O3 type structures (defined by Delmas^[^ ^26^ ^]^) inserted in Figure 2a,b are obtained by VESTA software.^[^ ^27^ ^]^ The discrepancies between the two structures are clearly exhibited. Na ions occupy two different triangular prismatic sites surrounded by AA and BB oxygen slabs in P2‐type structure. In contrast, Na ions locate the center of three different octahedrons surrounded by AC, BA, and CB oxygen slabs in O3 type structure. The peaks of NFM are indexed well to a hexagonal phase with P6_3_/mmc space group, except a small peak near 34^o^ which results from impurity. The peaks of NLFM can be assigned to the incorporated phases combining both P2 type and O3 type structure, coinciding well with the hexagonal lattice (space group: P6_3_/mmc) and rhombohedral lattice (space group: R‐3m). The P2 type and O3 type structures are isostructural with Na_0.7_MnO_2.05_ (JCPDS: 27‐0751) and *α*‐NaFeO_2_ (JCPDS: 20‐1115). The lattice parameters for NFM are *a* = *b* = 2.934 Å, *c* = 11.21 Å. For NLFM, the calculated lattice parameters of P2 part are refined as *a* = *b* = 2.884 Å, *c* = 11.04 Å, and O3 part are fitted for *a* = *b* = 2.92 Å, *c* = 16.43 Å. Moreover, there are small peaks around 20^o^ which belong to the superlattice peaks originating from the ordered arrangement between Li and transition metals.^[^ ^28^, ^29^, ^30^ ^]^

It is widely reported that Mn/Fe‐based layered cathodes are mostly sensitive to air. To contrast the stability against ambient air, the two materials were exposed to air for one month. The comparison of XRD patterns is shown in Figure 2c (full range patterns are shown in Figure S5, Supporting Information). NLFM shows an unchanged pattern even after exposing to air for 1 month. The peaks located between 16^o^ and 17^o^ are assigned as (002) for P2 type and (003) for O3 type, respectively. NFM, however, shows an additional peak near (002) peak, marked by ∗. Moreover, (002) peak has a slight shift to the lower angle, indicating the enlarged interlayer spacing. Meanwhile, the broadening peak width and decreased peak intensity also evidence the structural instability of NFM against air. Note that CO_2_ component in the atmosphere may attack NFM and cause the structure breaking of NFM.^[^ ^14^ ^]^ Further, the materials were soaked in deionized water for a long time of one week followed by drying at 60^o^ (right side of Figure 2c). Similar to the material exposed to air, the XRD pattern of water‐soaked NLFM coincides well with the pristine one. Regarding NFM, the structural degeneration is more serious, represented by a new obvious broad peak near 15^o^. The whole pattern is different from the pristine one, indicating the structure has been destroyed by the extraction of Na and the insertion of water between the layers. Moreover, the SEM (scanning electron microscopy) images of NFM and NLFM with pristine and water‐soaked samples are shown in Figure S6 (Supporting Information) for comparison. For NFM, new small particles appear on the layered blocks after soaking in water, confirming the emergence of a new phase. By contrast, both pristine and water‐soaked samples show layered morphologies for NLFM, consistent well with the XRD patterns. The results demonstrate that NLFM has excellent stability against both ambient air (CO_2_ etc.) and water, as shown in the schematic diagram in Figure 2d.

**Figure 3:**
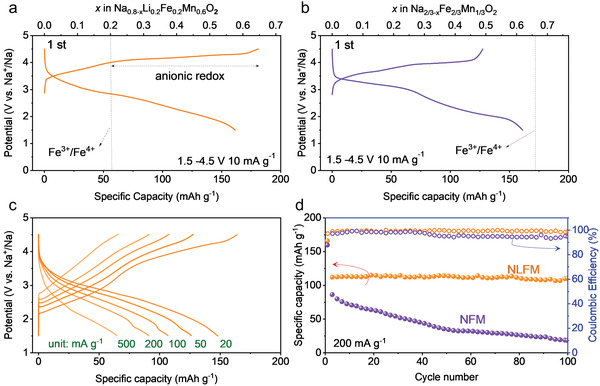
Comparison of electrochemical performances between NLFM and NFM. a) Typical initial charge‐discharge cycle of NLFM with a potential window of 1.5−4.5 V at 10 mA g^−1^. b) Typical initial charge‐discharge cycle of NFM with a potential window of 1.5−4.5 V at 10 mA g^−1^. c) Charge–discharge curves of NLFM at various current densities. d) Cycling performance of the two electrodes at 200 mA g^−1^ with coulombic efficiency.

The differences in electrochemical performances between NLFM and NFM are shown in **Figure** **3**. The initial charge‐discharge profile of NFM and NLFM is performed (Figure 3a,b), with a galvanostatic model and potential window of 1.5 −4.5 V under the current density of 10 mA g^–1^. The first charge curve can be divided into two parts, separated by the vertical dot line that represents the capacity based on Fe^3+^/Fe^4+^ (Figure 3a). The specific capacity of this first charge process is 181.1 mA h g^–1^, corresponding to 0.65 Na^+^ per formula extracted from the lattice. Note that Mn^4+^ cannot be oxidized to a higher valence in an octahedral environment.^[^ ^31^ ^]^ The capacity based on cationic redox process (active Fe^3+^) is only 55 mA h g^–1^, corresponding to 0.2 Na^+^, which means the excess capacity (beyond cationic redox process) is likely derived from anionic redox process. Moreover, Li substitution conceives the possibility of anionic redox and the corresponding plateau near 4 V is analogous to the typical Li‐rich cathodes.^[^ ^32^ ^]^ The results are consistent well with the DFT calculations. Details of the charge compensation mechanism will be discussed further in the next part. It is noted that the valences of TMs and structures will affect the electrochemical performance of Li‐substituted Fe‐ and/or Mn‐based layered cathodes.^[^ ^33^, ^34^ ^]^ As for NFM in Figure 3b, the first charge capacity is lower than the theoretical capacity based on Fe^3+^/Fe^4+^. It is obvious that the first discharge capacity is larger than the charge process, which is reasonable because NFM is a Na‐deficient material and Mn can be reduced during the discharge process. The remarkable comparison of derived d*Q*/d*V* profiles is shown in Figure S7 (Supporting Information). There are two peaks during the charge process in NFM, indicating the oxidation process of Fe with phase transition which will be discussed in the following part. As for NLFM, one extra peak appears at a high potential which is similar with Li_2_MnO_3_‐based Li‐rich materials,^[^ ^35^ ^]^ coinciding well with DFT results. The lower potentials marked by dotted rectangle represent Mn redox process. It is notable that Mn redox peak in NFM is relatively strong compared with Fe redox peak. This indicates an intense participation of Mn redox in charge compensation, which is adverse to structural stability.

The rate performance is naturally displayed between the two materials (Figure S8, Supporting Information). The current density of the initial 10 cycles is 20 mA g^–1^, followed by the subsequent current densities of 50, 100, 200, and 500 mA g^–1^ for every 10 cycles. The first discharge capacities for NLFM at each current density are 165, 128, 108, 89, and 64 mA h g^–1^, respectively. In contrast, the values for NFM are 154, 106, 81, 51, and 19 mA h g^–1^, respectively. Apparently, NLFM possesses impressive rates of performance and a superior recovery capability when the large current density returns to a small one (136 mA h g^–1^ for NLFM and 103 mA h g^–1^ for NFM when the current density recovers to 20 mA g^–1^). The corresponding charge‐discharge curves are shown in Figure 3c and Figure S9 (Supporting Information). At high current density of 200 mA g^–1^, the discharge capacities of initial cycles for the two materials are comparable while the cycling stability shows quite remarkable disparity (Figure 3d; Figure S10, Supporting Information). The electrodes were tested with a low current density in the first cycle for their activation, followed by a high current density of 200 mA g^–1^. The capacity of NLFM and NFM after 100 cycles are 109.6 mA h g^–1^ and 17.8 mA h g^–1^ with the capacity retention of 98.34% and 11.97%, respectively. The coulombic efficiency of NLFM is almost larger than 99.0% with a stable tendency compared with the gradually decreased behavior in NFM. The results demonstrate NLFM has improved electrochemical performance compared with NFM in the applied current density. Anionic redox here, exhibits a positive effect on the electrochemical performances.

As is well known, the kinetics takes a prominent role in the electrochemical performance. To explore the dynamic difference between the two materials, GITT (galvanostatic intermittent titration technique) was performed for one cycle with the potential window of 1.5–4.5 V (Figure S11, Supporting Information). The cells were conducted with the current density of 10 mA g^–1^ for 1 h followed by 5 h rest to approach the quasi‐equilibrium potential (circle dot line). This procedure was repeated until the end of the cycle. Na^+^ diffusion coefficient (D_Na+_) therefore can be decided by Fick's second law of diffusion. Details about the equation and calculations were introduced in our previous work^[^ ^18^ ^]^ and also shown in Figure S12 (Supporting Information). The calculated D_Na+_ of NLFM which ranges from 10^–12^ to 10^–10^ cm^2^ s^–1^, is superior to that of NFM ranging from 10^–14^ to 10^–10^ cm^2^ s^–1^. Bounded by the potential of 4.0 V, the charging process can be divided into two parts. The value of D_Na+_ for both NLFM and NFM is approximate in the initial part before 4.0 V, demonstrating a similar oxidized process. During the second part beyond 4.0 V, a plateau emerges accompanied by the slower kinetics. However, the plateau is different between the two materials. For NLFM, this plateau is related to the anionic redox process, while the plateau of NFM reflects the phase transition. It is apparent that the phase transition in NFM with slow kinetics impairs the rate capability, in accordance with Figure S9 (Supporting Information). Note that unlike the sluggish kinetics at the high‐potential plateau in typical Li‐rich materials, the corresponding anionic redox process is accelerated and the kinetics is improved due to Li substitution in NLFM, benefitting from the limited oxygen loss and stable structure (without detected phase transition), which will be discussed below.

**Figure 4:**
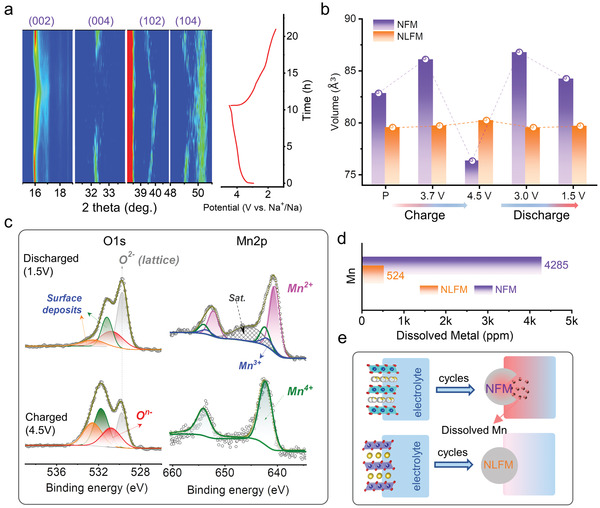
Structure evolution of the cathodes. a) In situ XRD color‐filled patterns of NLFM for the first cycle accompanied by the corresponding charge–discharge curve. b) Evolutions of cell volume for NFM and NLFM during the first cycle. The data are obtained after refining the corresponding XRD patterns. c) XPS results of O1s and Mn2p spectra for charged (4.5 V) and discharged (1.5 V) NLFM electrodes. d) Concentration of dissolved metal in two electrodes obtained from electrolyte after 100 cycles. e) Schematic diagram of dissolved Mn with cycles.

To investigate the structural evolution of the two materials, in situ and ex situ XRD were performed. **Figure** **4**a shows the in situ XRD color‐filled patterns for the first cycle of NLFM accompanied by the corresponding charge‐discharge curve. The whole range of XRD patterns is displayed in Figure S13 (Supporting Information). The symbol ∗ represents the impurity from the in situ model, which is confirmed by comparing the different states of XRD patterns, as shown in Figure S14 (Supporting Information). The (002) peak firstly shifts to a lower angle, indicating the evolutions of *c* axis resulting from the extraction of Na^+^. The tendency persists until charging to ≈4.0 V, consistent with the result of sodium ion diffusion coefficient. Then (002) shifts back to a higher angle when charging to higher voltage. The two inverse behaviors during the charge process agree well with the in situ XRD results of Li_1.2_Ni_0.2_Mn_0.6_O_2_, which also indicates oxygen redox.^[^ ^17^ ^]^ The (002) peak returns to its original position after the discharge process, demonstrating good reversibility. Moreover, the in situ XRD patterns for the second cycle are also displayed in Figure S15 (Supporting Information). Similarly, NLFM exhibits remarkable structural stability, more importantly, without phase transition. In contrast, NFM shows an additional peak after the charging process, locating at ≈18^o^ (Figure S16, Supporting Information). The new peak indicates the phase transition from P2 to O2 or Z phase, which is common in P2 type materials.

Moreover, the volume evolutions of two electrodes during the first cycle are compared in Figure 4b, calculated from *a* and *c* lattice (Figure S17, Supporting Information) after refining the cell parameters. The key potential points are selected based on their corresponding d*Q*/d*V* curves. Obviously, NFM suffers continuous volume changes, with a value of 7.9% during charge and 10.4% during discharge. The reduplicative volume change deteriorates the structural stability and further results in capacity fade. In contrast, NLFM exhibits only 0.85% volume change during cycle, which is far less than that of NFM, profiting from Li substitution. Actually, the distance of oxygen slabs in NFM expands during charge because of the increasing repulsion from the extraction of Na^+^. The structure cannot maintain P2‐type stacking with slight Na^+^, inducing the phase transition for stability. On the contrary, the distance of oxygen slabs will not increase sustainably in NLFM stemming from the oxidation of oxygen, which decreases the repulsion and stabilizes the structure.^[^ ^19^ ^]^ The two‐phase transition behavior well explains the plateau around 4.0 V in the first cycle for NFM in Figure 3b and d*Q*/d*V* curves (Figure S7), which demonstrate that phase transition in NFM induces the sluggish kinetics and eventual failure of the electrochemical performance, corresponding to the GITT results. On the contrary, NLFM owns the structural stability and reversibility without phase transition, leading to its superior electrochemical behavior. It is noted that the oxygen redox usually leads to gaseous oxygen loss, damaging the stability of the structure. To confirm the oxygen evolution in NLFM, differential electrochemical mass spectrometry (DEMS) was applied. Results show that the oxygen loss is very limited in NLFM in the first cycle and cannot be detected in subsequent cycles (Figure S18, Supporting Information).

Then, the charge‐discharge mechanism of the cathodes was investigated by XPS and sXAS. XPS results of NLFM show the evolutions for redox states of O and Mn (Figure 4c). When charging to 4.5 V, the electrode shows a new peak located at ≈531 eV, representing the oxidized oxygen which is well consistent with other works.^[^ ^36^, ^37^, ^38^ ^]^ The results coincide with that of in situ XRD patterns. Additionally, it is noted that, after discharging the electrodes to 1.5 V, Mn2p spectrum of NLFM propagates a small peak at ≈640.6 eV, which is assigned as Mn^2+^ (the spectra of NFM is compared in Figure S19, Supporting Information). Furthermore, inductive coupled plasma emission spectroscopic analyzer (ICP) for the electrolyte was performed to measure the Mn content dissolved from NLFM and NFM electrodes, after 100 cycles. Results (Figure 4d) demonstrate that the concentration of dissolved Mn in NFM is eight times greater than that in NLFM. It implies that overexploitation of Mn in NFM generates more serious Mn dissolution, which weakens the structural stability. By contrast, this overexploitation is inhibited in NLFM by the charge compensation of anionic redox (Figure 4e) which realizes a trade‐off between anionic redox and cationic redox. Anionic redox acts as a key factor to balance the charge compensation, and avoid the loss of active material as well as structural distortion.

**Figure 5:**
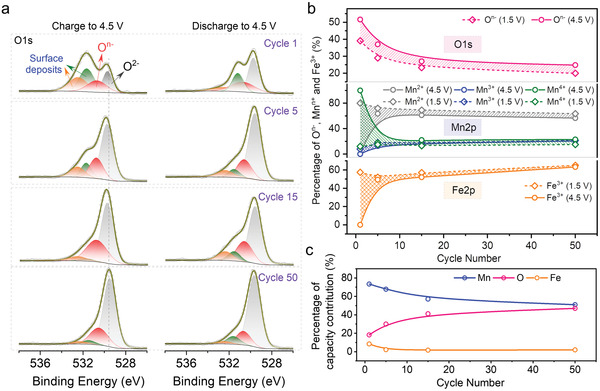
Ex situ XPS analysis of the oxygen evolution. a) Fitted curves of O1s spectra for charged (4.5 V) and discharged (1.5 V) NLFM electrodes over cycling. b) Percentage of various valences of O, Mn, and Fe in O1s, Mn2p, and Fe2p spectra respectively for charged (4.5 V) and discharged (1.5 V) NLFM electrodes over cycling. The values are calculated by the ratio of one specific integrated area to the total integrated element. For instance, integrated O*^n^* ^−^ to the integrated (O^2−^ and O*^n^* ^−^) gives the ratio of O*^n^* ^−^. The shaded area represents the reversible capacity contribution. c) Capacity contribution of Mn, O, and Fe over cycling. The calculated details are shown in Supporting Information.

In addition, the capacity contribution of three electrochemically active elements (O, Mn, Fe) is also estimated. **Figure** **5**a exhibits the XPS results of O1s spectra over cycling (Mn2p and Fe2p are shown in Figures S20 and S21 in Supporting Information, respectively) for charged (4.5 V) and discharged (1.5 V) NLFM electrodes. The cells were tested under 10 mA g^−1^ for the first cycle and then 200 mA g^−1^ for subsequent cycles. Until the aimed cycle, the current density was returned to 10 mA g^−1^. It is noted that O^n−^, Mn^2+/3+^, and Fe^3+^ exist whether in charge or discharge process (Figure 5b), which mean only partial element delivers electrochemical activity. The capacity contribution of three elements is estimated based on the active part (shaded area in Figure 5b) with the result shown in Figure 5c (details in Supporting Information). Manganese dominates the capacity contribution in the initial cycles, followed by oxygen. The evolution tends to equilibrium after 20 cycles, where the contribution of iron can be neglectable while Mn and O display almost equal capacity contribution. To deeply investigate the valence evolution of Mn, the electrodes were further etched (to 300 nm) and tested using XPS, with the results shown in Figure S22 (Supporting Information). All the electrodes mainly contain Mn^3+^ and Mn^4+^ states with limited Mn^2+^. It should be noted that Ar^+^ etching will induce the reduction of manganese to a certain degree, with the generation of Mn^2+^. The results show the sustainably reversible capacity contribution of anionic redox and stable chemical environment of the interface, preventing Mn from overexploitation and improving the stability of NLFM electrodes.

The sXAS for O K‐edge of both NFM and NLFM were employed to further investigate the anionic behavior (Figure S23, Supporting Information). Here we focus our attention on the pre‐edge features because of the valuable information about the valence change of transition metals and oxygen holes. The peak increases from “P” (pristine) to “C1” (charge to 60 mA h g^−1^), demonstrating the oxidation of transition metal. The oxidation process generates more holes in 2p‐3d hybrid states, leading the peak to increase.^[^ ^39^ ^]^ Additionally, as shown in previous works about Li‐rich materials, the peak near 531 eV can be assigned to O‐hole creation in the non‐bonding O 2p states.^[^ ^40^, ^41^ ^]^ Thus, the integrated pre‐edge peak intensity ranging between 528−532 eV is reasonably considered to reveal the information of oxygen oxidation.^[^ ^39^, ^42^ ^]^ The integrated pre‐edge peak intensity exhibits visualized results of different states of electrodes about the oxygen hole distribution corresponding to the anionic redox (Figure S23c,d in Supporting Information). Compared with the pristine state, the integrated intensity increases ≈17% for NFM after charging (C2), which is far below the value of ≈80% for NLFM, confirming the high active anionic redox process in NLFM.^[^ ^39^ ^]^ The result consists well with that of electrochemical performance, suppressed phase transition in in situ XRD and the variation of elemental valence from XPS.

**Figure 6:**
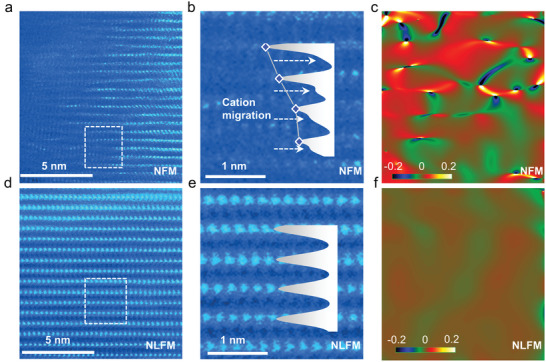
STEM imaging of NFM and NLFM after 100 cycles. a) The high‐angle annular dark‐field (HAADF)‐STEM image of NFM with the selected area electron diffraction (SAED) pattern. b) Enlarged HAADF‐STEM image obtained from a, with the extracted intensity profile. c) Corresponding geometric phase analysis (GPA) patterns of a. d) The HAADF‐STEM image of NLFM with the SAED pattern. e) Enlarged HAADF‐STEM image obtained from d, with the extracted intensity profile. f) Corresponding geometric phase analysis (GPA) patterns of d.

To reveal the structural evolution of the cycled cathodes STEM with HADDF model was utilized. The bright‐dots in **Figure** **6**a,c correspond the TM‐ions (Fe and Mn), accompanied by corresponding fast Fourier transform for NFM and NLFM, respectively. The arrangement of TMs in NFM is fluctuant together with the formation of massive vacancies. While TMs arrange in a perfect order for NLFM. Figure 6b,e show the enlarged images selected in the rectangle of Figure 6a,d, respectively, with the extracted intensity profile. The intensity profile shows obvious difference, represented by a gradually decreased intensity in NFM and an equivalent height in NLFM. It is noted that there are increasing signals between TM layers in Figure 6b labeled by white arrows, indicating cation migration. The TM vacancy and cation migration coincide well with the ICP results of Mn dissolution. In contrast, there are no perceptible deficiencies for NLFM, demonstrating negligible Fe migration and Mn dissolution. Moreover, corresponding geometric phase analysis (GPA) patterns have notable difference between two cathodes (Figure 6c,f), indicating lattice stress in electrodes, which shows apparently heterogeneity for NFM and uniformity for NLFM. The unbalanced lattice stress existed in NFM easily induces phase transition, which corresponds well with the in/ex situ XRD results.

In summary, we report a layered oxide Na_0.8_Li_0.2_Fe_0.2_Mn_0.6_O_2_ (NLFM) with anion–cation synergetic redox to realize reduced lattice strain and splendid electrochemical performance. Long‐term structural stability during continuous sodium extraction and intercalation is achieved by its prominent stability against moist air, solid solution behavior with 0.85% volumetric change as well as the negligible manganese dissolution and iron migration. Our work will take a step forward to understand anionic redox chemistry and pave the way for designing high‐stable cathode materials contributing to high‐performance sodium‐ion batteries.

## Conflict of Interest

The authors declare no conflict of interest.

## Supporting information

Supporting Information

## Data Availability

The data that support the findings of this study are available from the corresponding author upon reasonable request.
