# Supplementary material for: Synergetic Anion–Cation Redox Ensures a Highly Stable Layered Cathode for Sodium‐Ion Batteries
Source: Adv Sci (Weinh). 2022 Apr 7;9(16):2105280. doi: 10.1002/advs.202105280 (PMC9165485; doi:10.1002/advs.202105280)
Supplement: Supplementary file 1 — Supporting Information [file ADVS-9-2105280-s001.pdf]

ADVANCED SCIENCE
Open Access

Supporting Information

for *Adv. Sci.*, DOI 10.1002/advs.202105280

Synergetic Anion–Cation Redox Ensures a Highly Stable Layered Cathode for Sodium-Ion Batteries

Xiang Li, Jialiang Xu, Haoyu Li, Hong Zhu, Shaohua Guo and Haoshen Zhou**

((Supporting Information can be included here using this template))

© 2022 Wiley-VCH GmbH

Supporting Information

Synergetic Anion-Cation Redox Ensures a Highly Stable Layered Cathode for Sodium-Ion Batteries

Xiang Li,[a,c] Jialiang Xu,[d] Haoyu Li,[a,b] Hong Zhu,[d] Shaohua Guo,[a,b*] Haoshen Zhou[a*]

[a] Center of Energy Storage Materials & Technology, College of Engineering and Applied Sciences, Jiangsu Key Laboratory of Artificial Functional Materials, National Laboratory of Solid State Microstructures, and Collaborative Innovation Center of Advanced Microstructure
Nanjing University
Nanjing 210093, China
[b] Shenzhen Research Institute of Nanjing University, Shenzhen 51800, China
[c] College of Chemistry, Zhengzhou University, Zhengzhou 450001, China
[d] University of Michigan-Shanghai Jiao Tong University Joint Institute
Shanghai Jiao Tong University
Shanghai 200240, China

Experimental procedure

Synthesis of $Na_{0.8}Li_{0.2}Fe_{0.2}Mn_{0.6}O_2$ and $Na_{2/3}Fe_{2/3}Mn_{1/3}O_2$:

$Na_{0.8}Li_{0.2}Fe_{0.2}Mn_{0.6}O_2$ (NLFM) powder was synthesized by solid-state reactions with Na_2CO_3 (Wako), Li_2CO_3 (Wako), Fe_2O_3 (Sigma-Aldrich) and MnO_2 (Wako). The starting materials were weighted and mixed at a ratio of Na:Li:Fe:Mn = 4.08:1.02:1:3 and grinded for half an hour with mortar and pestle. Then the mixed powder was pelleted and calcined at 700 ^{o}C in air for 15 h, subsequently followed by calcined at 850 ^{o}C in air for 20 h and naturally cooled to room temperature. For $Na_{2/3}Fe_{2/3}Mn_{1/3}O_2$ (NFM), the procedures are the same as $Na_{0.8}Li_{0.2}Fe_{0.2}Mn_{0.6}O_2$, with a different calcined process by 980 ^{o}C in air for 15 h.

Electrochemical tests:

2032 coin-type cells were used for electrochemical measurements. The electrodes for both NLFM and NFM are consisted of active material, acetylene black, and polytetrafluoroethene (PTFE, 12 wt.%) binder with the weight ratio of 80:10:10. 1 M $NaPF_6$ in EC/DEC (ethylene carbonate/diethyl carbonate) was prepared as the electrolyte and a glass fiber film was employed as separator. The galvanostatic charge-discharge tests were performed by using a Hokuto Denko HJ1001SD8 battery tester at different conditions.

Characterizations:

The morphology of the powder was observed using scanning electron microscopy (SEM, TOPCON DS-720 instrument). XPS was characterized by a Thermo Fisher Scientific Model Kα spectrometer

equipped with Al Kα radiation (1486.6 eV). Before XPS test, the sample was transferred into an Ar glove box and sealed in an airtight container to avoid the exposure with the humidity.

Estimation of relative capacity contributions using XPS spectra

The specific discharge capacity of NLFM is ~ 160 mAh g^{-1} for the first cycle, indicating 0.6 e^{-} transfer. Considering Mn only, there are 80% Mn^{2+} and 8% Mn^{3+} calculated by Mn2p XPS spectra. Given that Mn are full of +4 valence after the first charge, Mn can deliver 1.008 e^{-} transfer (0.6×80%×2+0.6×8%), which is far larger than 0.6 e^{-}. It is noted that XPS is a surface-sensitive technique, gathering the information from several nano-meters, which leads to the deviation with the actual situation. Herein, the capacity contribution of O, Mn, and Fe is just calculated from the XPS information. After the first charge, the proportion of O^{n-} in total oxygen is 51.6% (O^{n-}/O). Both Mn and Fe are of their highest valence (+4 and +3, respectively). After the first discharge, the proportion of electrochemically active elements are 39.1% for O^{n-} ($O^{n-}/(O^{n-}+O^{2-})$), 80% for Mn^{2+} ($Mn^{2+}/(Mn^{2+}+Mn^{3+}+Mn^{4+})$), 8% for Mn^{3+} ($Mn^{3+}/(Mn^{2+}+Mn^{3+}+Mn^{4+})$), and 57.4% for Fe^{3+} ($Fe^{3+}/(Fe^{3+}+Fe^{4+})$). Therefore 12.5% O^{n-} (51.6% − 39.1%) is reversible for the first cycle. The capacity contribution of O, Mn, and Fe during the first cycle is assumed to be proportional to 0.25 (2×12.5%×1, when n = 1) : 1.008 (mentioned above) : 0.1148 (0.2×57.4%). That is 18.2%, 73.4%, 8.4% for O, Mn, and Fe respectively. The capacity contribution of the elements for subsequent cycles is also calculated by this method. Note that the method is based on some speculations which is just

an estimation. Moreover, the capacity contribution has reference value on analyzing the surface evolution which has relationship with the structural stability.

Statistical Analysis:

Calculations Method

Our approach is to use the projector augmented wave (PAW)[1, 2] method density functional theory (DFT) calculations within the Vienna ab initio simulation package.[3] The generalized gradient approximation (GGA) and Perdew–Burke–Ernzerhof (PBE)[4] exchange functional was used. The optimized ground state configurations were performed at constant volume using a $Na_{2/3}Li_{1/6}Fe_{1/6}Mn_{2/3}O_2$ with 24 formula units (f.u.) ($Na_{1/3}Li_{1/6}Fe_{1/6}Mn_{2/3}O_2$ with 18 f.u.) supercell and a Gamma grid with 3 × 3 ×2 (4×4×4 for $Na_{1/3}Li_{1/6}Fe_{1/6}Mn_{2/3}O_2$) k-point sampling and a cut-off energy of 600 eV for structures optimization. To study the more accurate redox activity, the calculations for DOS and COHP implemented in LOBSTER[5, 6] were performed with a Gamma grid with 6 × 6 × 4 k-point sampling.[5] The energy barriers of Na migration paths were calculated in the CAVD (crystal structure analysis by voronoi decomposition)+BVSE (bond valence site energy) method.[7] We applied the GGA + U method to calculate the nature of Fe and Mn 3d states, choosing 4.0 eV as the U value for Fe and Mn. The convergence criterions of the energy and force were 10^{-5} eV/atom.

DFT calculations

A series of 1004 structures containing up to 24 (P2) formula units (f.u.) with varying Na vacancies

patterns are generated for this study by pymatgen code[8] and Ewald summary Energy.[9] Then, we calculated the DFT energy of the first 50 structures and chose the three lowest energy structures, named NLFM_1, NLFM_2, NLFM_3. The Na1 arrangement in the P2 phase of NLFM has two different patterns (zigzag and flower)[10, 11], as shown in **Figure S2**. The red atoms are Na1 and the yellow atoms are Na2. The Na1 in three NLFM structures has a similar pattern but Na2 has different patterns. So, the Na/vacancy in $Na_{2/3}Li_{1/6}Fe_{1/6}Mn_{2/3}O_2$ composition might have different patterns in NLFM.

Structure

We build the structural models of NLFM in two steps according to the proportions of the elements obtained in the experiment. The construction of NFM is from Katcho's work.[12] There are two nonequivalent prismatic Na sites in the experimental results of NLFM. Na1 in the 2*b* site and Na2 in the 2*d* site are in the ratio of 1:3. Lithium, Iron, and Manganese are located in octahedral sites of the same layer. The 3×3×1 $NaLi_{1/6}Fe_{1/6}Mn_{2/3}O_2$ probable supercells were built and enumerated with all the structures shown in **Figure S1**a. We choose the lowest Ewald summation energies[9] as the stable structure. The Lithium, Iron, and Manganese with the 1:1:4 compositions in honeycomb type was shown in Figure S1b.

The $Na_{2/3}Li_{1/6}Fe_{1/6}Mn_{2/3}O_2$ with 24 formula units (f.u.) probable supercells were built in **Figure S2**a. An Ewald preconditioning has been applied to the configurations and a series of 1004 structures

with varying Na/vacancy patterns were generated for this study. We calculated the DFT energy of the first 50 structures and chose the three lowest energy structures. The Na1 arrangement in the P2 phase of NLFM is the zigzag pattern and has two different patterns shown in Figure S2b. The red atoms are Na1 and the yellow atoms are Na2. The Na1 has a similar pattern but Na2 has different patterns in three NLFM structures. So, the Na/vacancy in $Na_{2/3}Li_{1/6}Fe_{1/6}Mn_{2/3}O_2$ composition might have different patterns in NLFM.

The Na position of P2/O3-$Na_{1/3}TMO_2$ was in Na2 site[10, 12]. The phase transition was observed in NLFM during the charging process from P2 to O3. The Na site was the same in P2/O3-$Na_{1/3}TMO_2$. We generate all nonequivalent Na/vacancy configurations for 18 f.u. supercells in **Figure S3**.

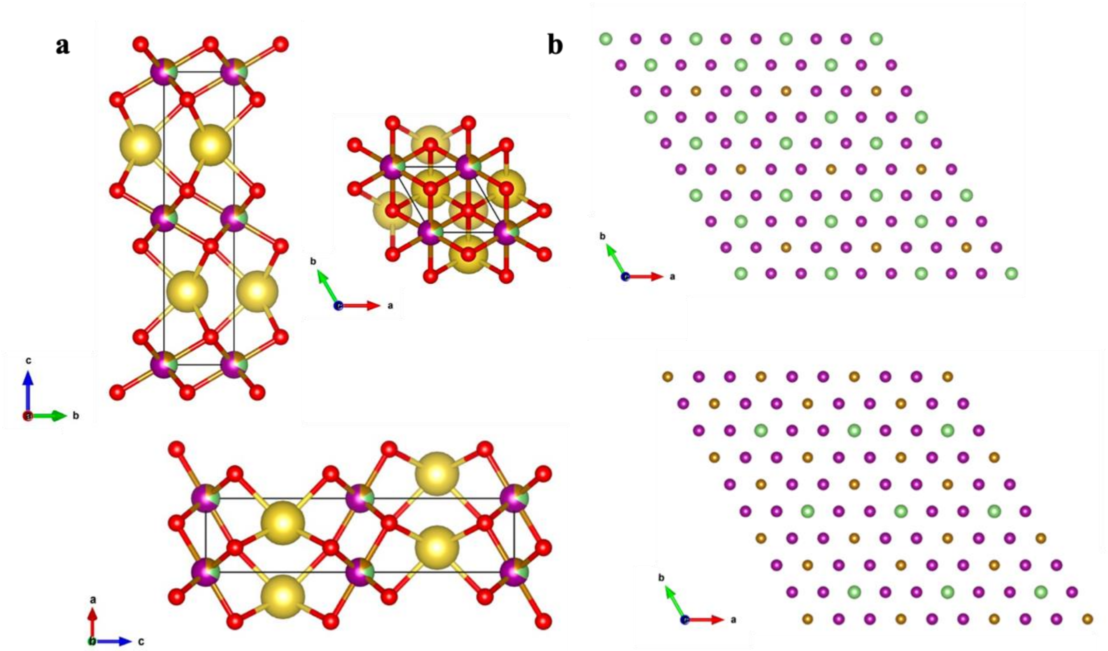

Figure S1. The structure of $NaLi_{1/6}Fe_{1/6}Mn_{2/3}O_2$ (a) Probable structure of $NaLi_{1/6}Fe_{1/6}Mn_{2/3}O_2$ and

(b) Transition metal layer of $Na_{1/3}Li_{1/6}Fe_{1/6}Mn_{2/3}O_2$.

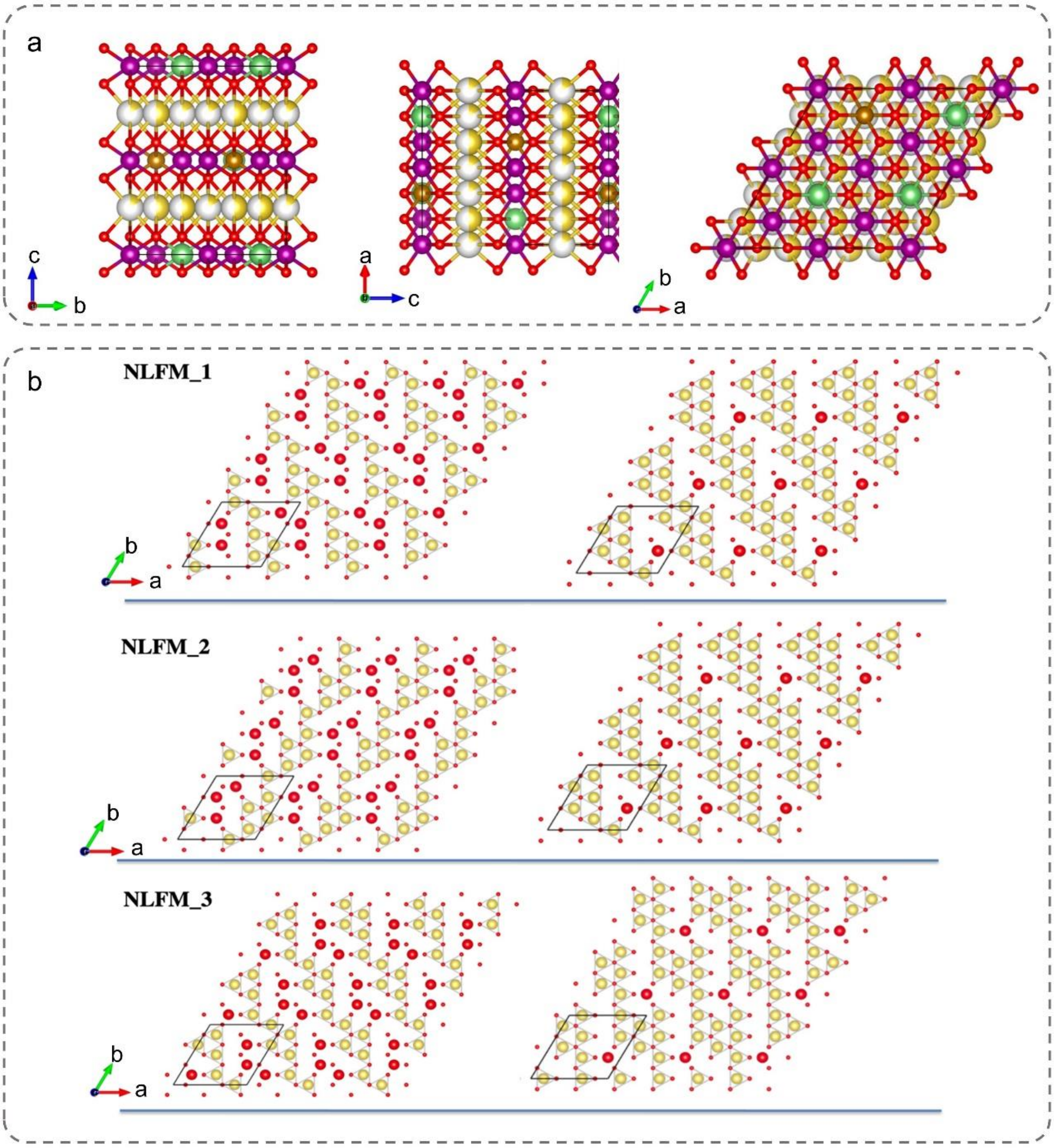

Figure S2. The structure of $Na_{2/3}Li_{1/6}Fe_{1/6}Mn_{2/3}O_2$ (a) Probable structure of $Na_{2/3}Li_{1/6}Fe_{1/6}Mn_{2/3}O_2$ and (b) Na pattern of $Na_{2/3}Li_{1/6}Fe_{1/6}Mn_{2/3}O_2$. Two Na layers of NLFM_1, NLFM_2 and NLFM_3. The left Na arrangement is the zigzag pattern and the right pattern is flower. The red atoms are Na1 and the yellow atoms is Na2.

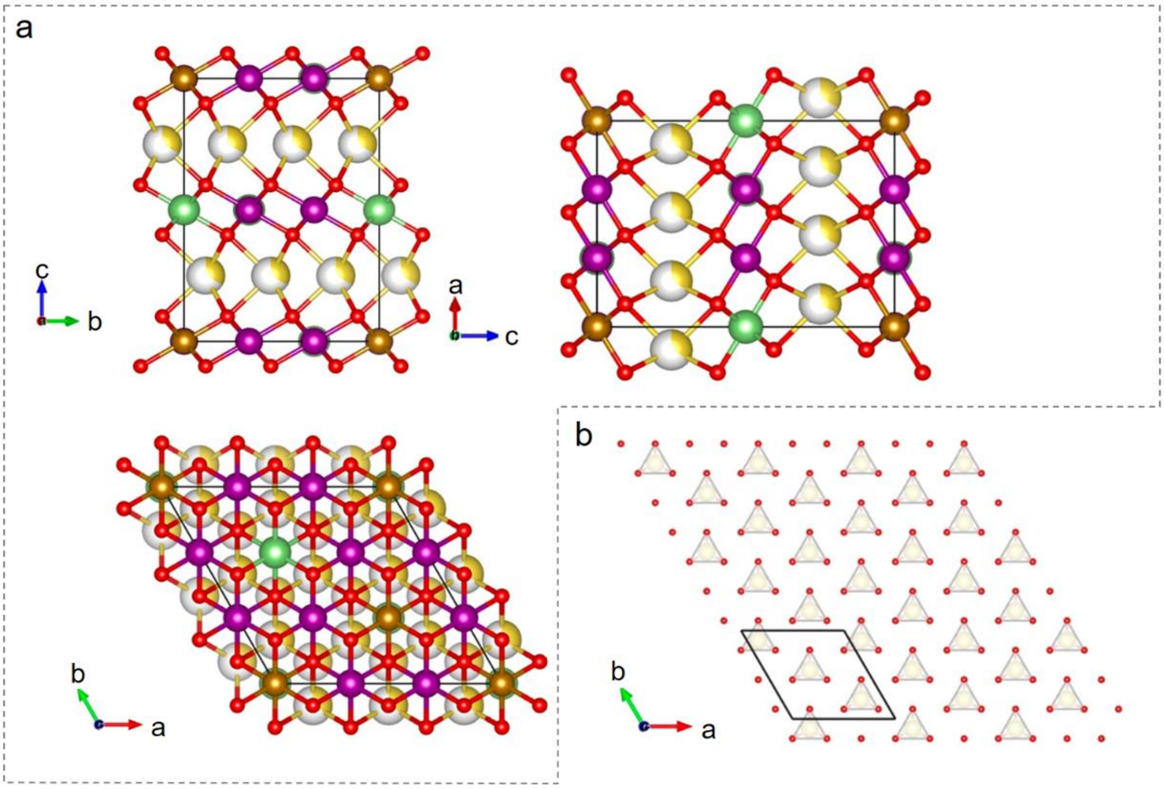

Figure S3. The structure of $Na_{1/3}Li_{1/6}Fe_{1/6}Mn_{2/3}O_2$. (a) Probable structure of $Na_{2/3}Li_{1/6}Fe_{1/6}Mn_{2/3}O_2$ and (b) Na pattern of $Na_{1/3}Li_{1/6}Fe_{1/6}Mn_{2/3}O_2$.

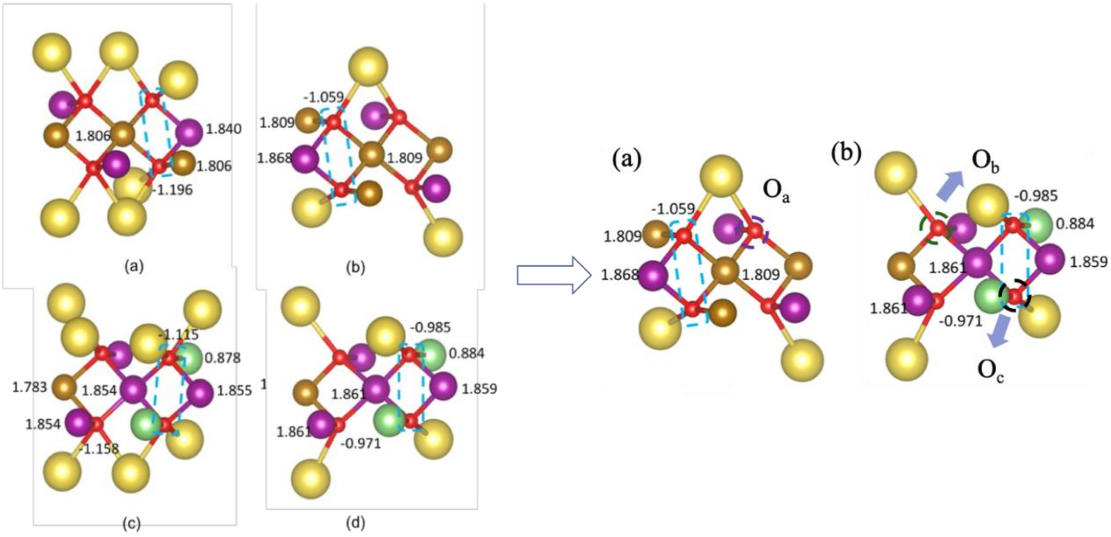

Figure S4. Details of different positions of oxygen in the crystal structure. (a) Half-charged NFM. (b) Half-charged NLFM. The O atom in color dash circle of purple, green and black represents O_a, O_b and O_c. The numbers near atoms are Bader charge of nearby atoms. The atom with color of yellow, green, brown, purple, and red represents Na, Li, Fe, Mn and O.

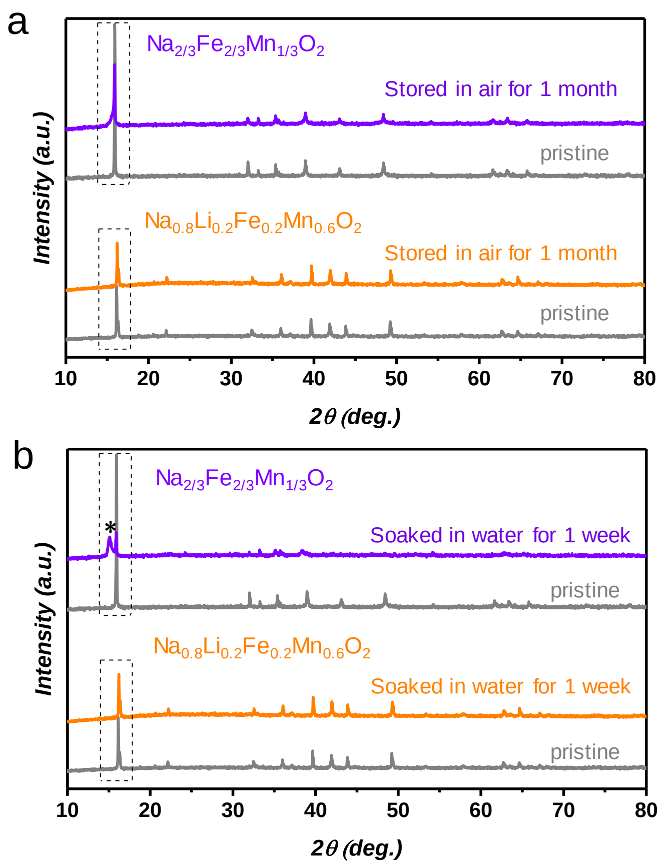

Figure S5. **a** Comparison of XRD patterns for NFM and NLFM after exposing the materials in air for one month. **b** Comparison of XRD patterns for NFM and NLFM after soaking the materials in water for one week.

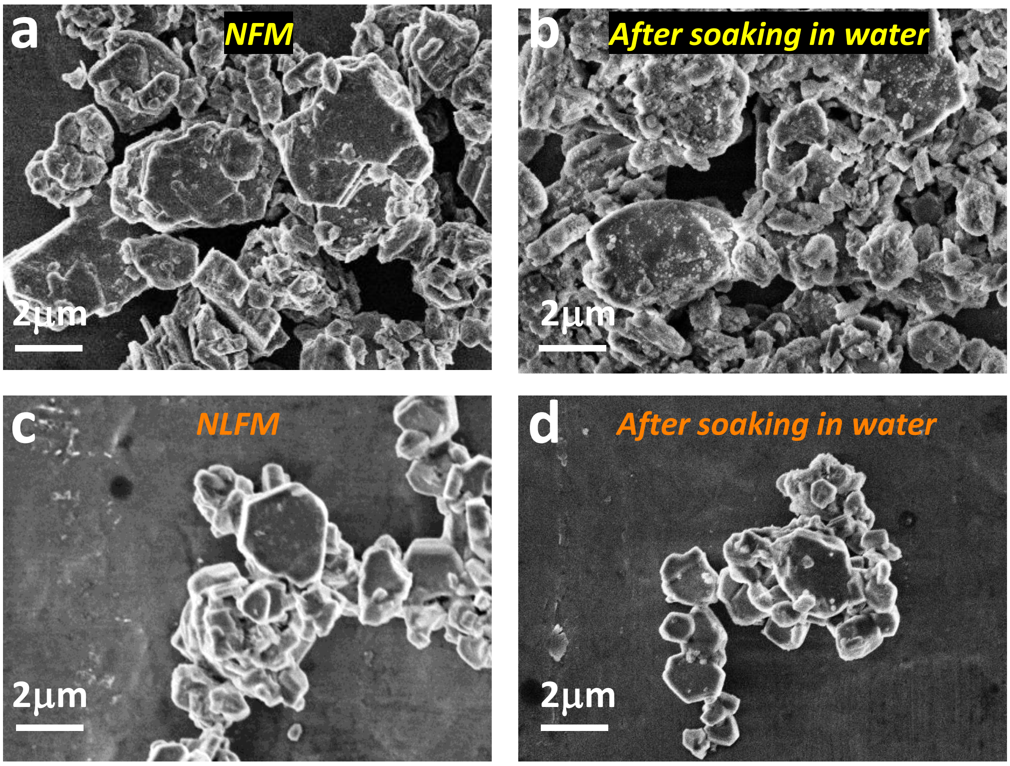

Figure S6. Comparison of SEM images between $Na_{2/3}Fe_{2/3}Mn_{1/3}O_2$ (NFM) and $Na_{0.8}Li_{0.2}Fe_{0.2}Mn_{0.6}O_2$ (NLFM). **a.** The pristine NFM. **b.** Water-soaked NFM for one week. **c.** The pristine NLFM. **d.** Water-soaked NLFM for one week.

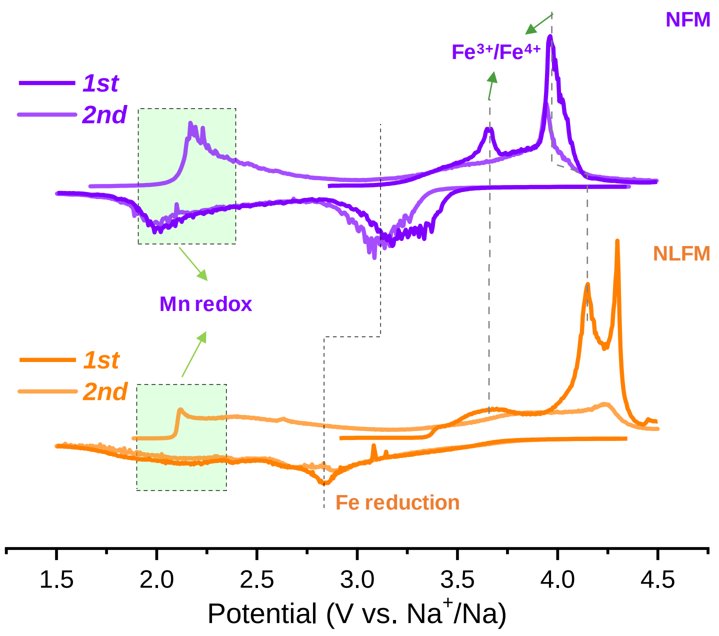

Figure S7. The corresponding dQ/dV curves of NLFM and NFM for the first two cycles at the current density of 10 mA g^{-1}.

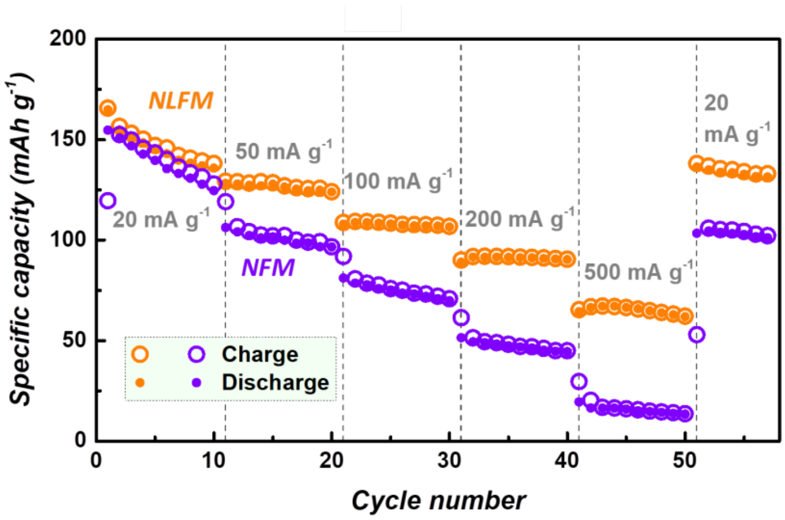

Figure S8. Rates performance of the two electrodes with the current densities of 20, 50, 100, 200 and 500 mA g^{-1}, respectively.

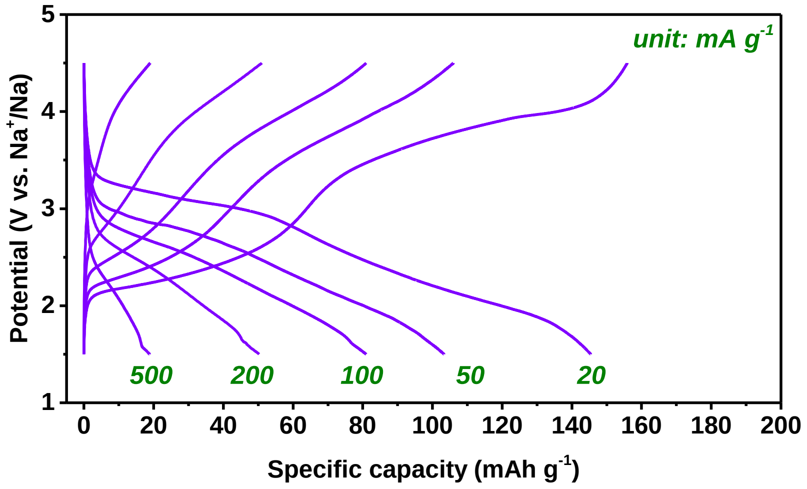

Figure S9. Charge-discharge curves of NFM at various current densities.

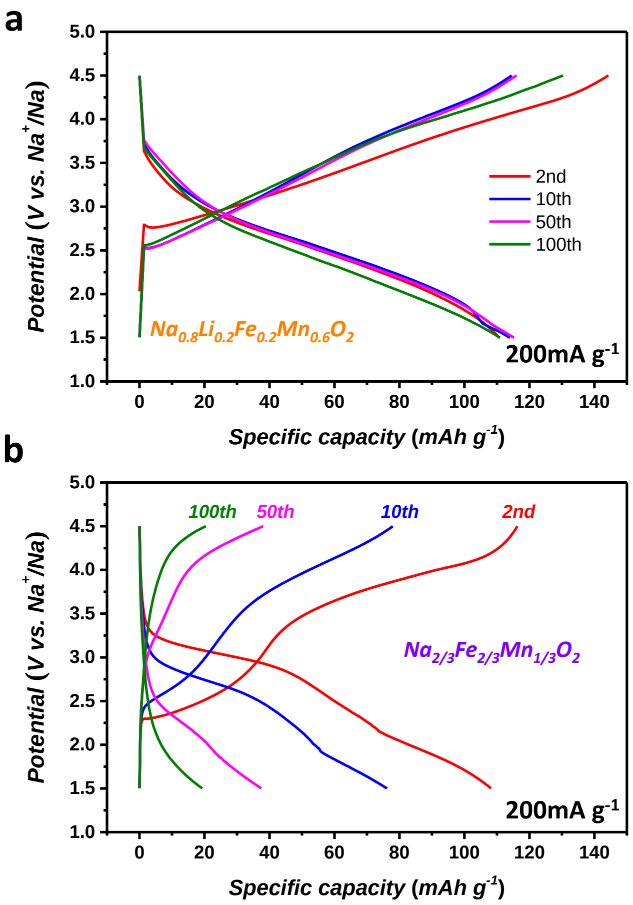

Figure S10. Charge-discharge curves for **a.** $Na_{0.8}Li_{0.2}Fe_{0.2}Mn_{0.6}O_2$ and **b.** $Na_{2/3}Fe_{2/3}Mn_{1/3}O_2$ at the current density of 200 mA g^{-1}.

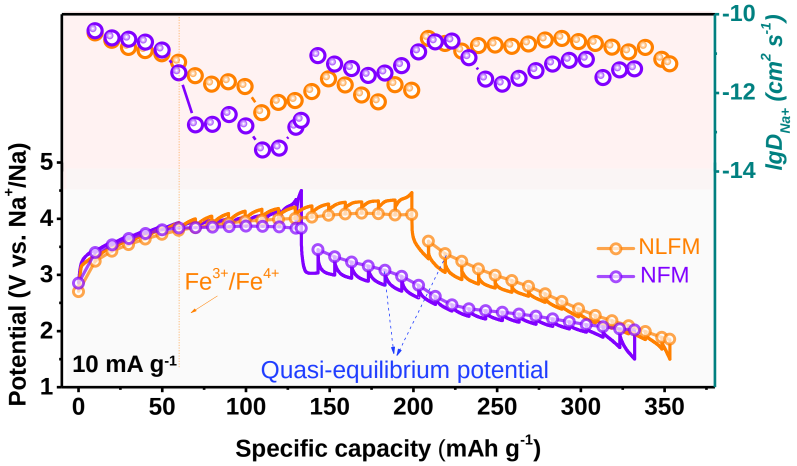

Figure S11. GITT profiles of the two electrodes for the first cycle at 10 mA g^{-1}, accompanied by the quasi-equilibrium potential (circle dot line) and calculated Na^+ diffusion coefficient.

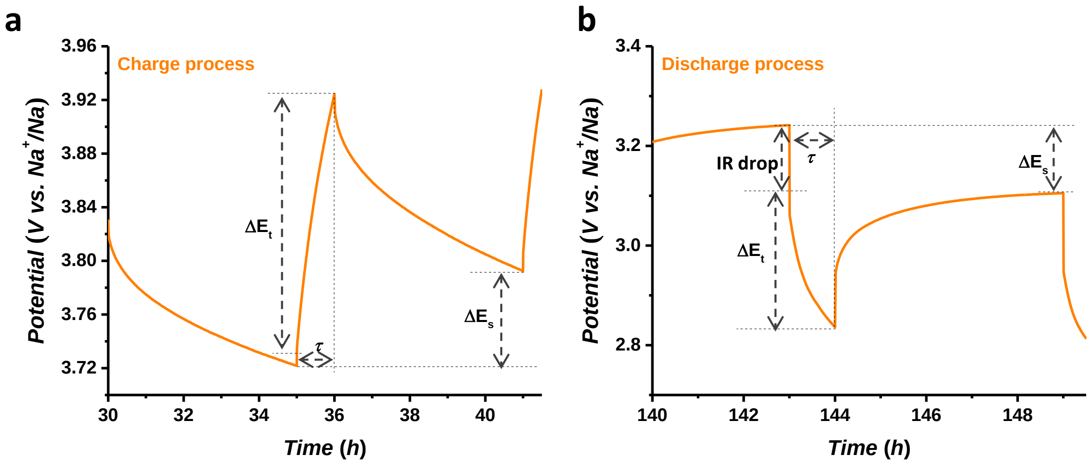

Figure S12. a. The potential profile for a single titration at 3.84 V during charge process with labelling the different parameters. **b.** The potential profile for a single titration at 3.20 V during discharge process with labelling the different parameters.

The D_{Na+} can be determined by inducing Fick's second law of diffusion based on some reasonable assumptions. Thus, the equation can be simplified to:

$$D_{\mathrm{Na}} = \frac{4}{\pi\tau}\left(\frac{\mathrm{n_m V_m}}{\mathrm{S}}\right)^2\left(\frac{\Delta \mathrm{E_s}}{\Delta \mathrm{E_t}}\right)^2$$

Where τ is the limited time period, n_m is the mole number of the electrode, V_m is the molar volume of the electrode, S is the area of electrode, ΔEs and ΔEt are the change in the steady state potential and the total change during the current flux by deducting the IR drop, respectively.

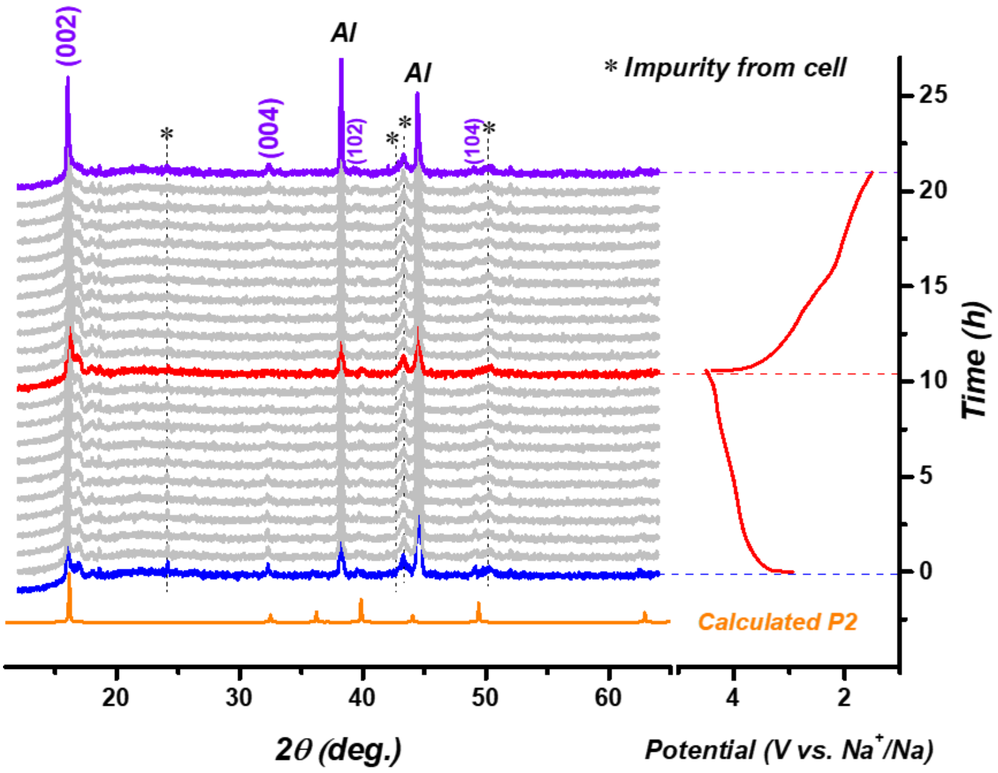

Figure S13. In situ XRD patterns of NLFM for the first cycle accompanied by the calculated pattern of P2 type.

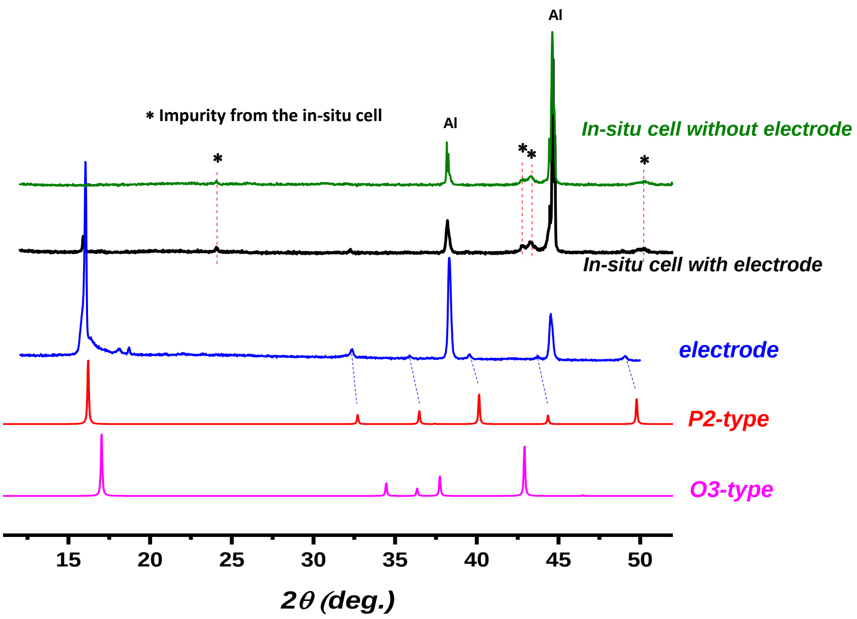

Figure S14. The comparison of XRD patterns between the different types of electrodes, accompanied by the calculated patterns of P2 type and O3 type. The impurity peaks are confirmed from the in-situ cell.

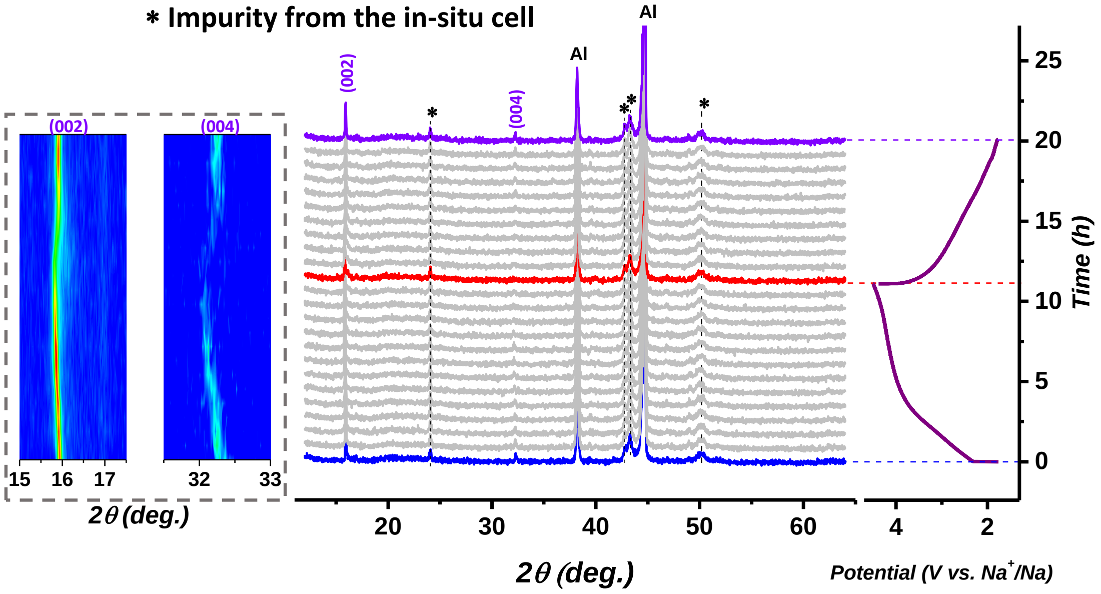

Figure S15. In situ XRD patterns of $Na_{0.8}Li_{0.2}Fe_{0.2}Mn_{0.6}O_2$ for the second cycle. The corresponding charge-discharge curve and color-filled patterns are also shown for comparison.

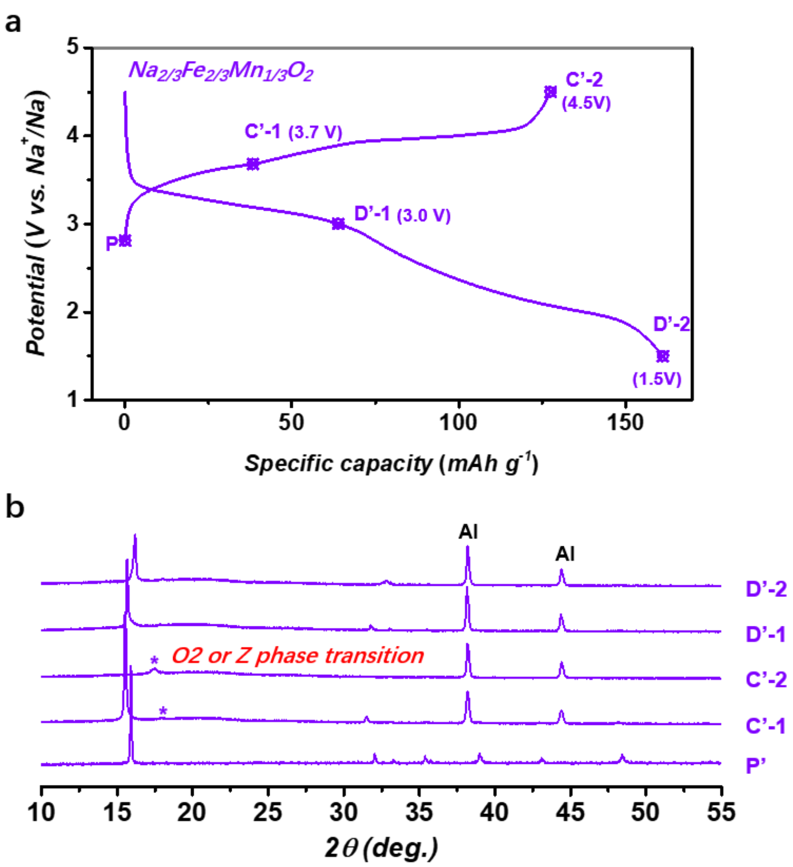

Figure S16. a. The first charge-discharge curve for $Na_{2/3}Fe_{2/3}Mn_{1/3}O_2$. **b.** Ex situ XRD patterns of $Na_{2/3}Fe_{2/3}Mn_{1/3}O_2$ for the first cycle. The denoted numbers in Figure S8b correspond to that of in Figure S8a.

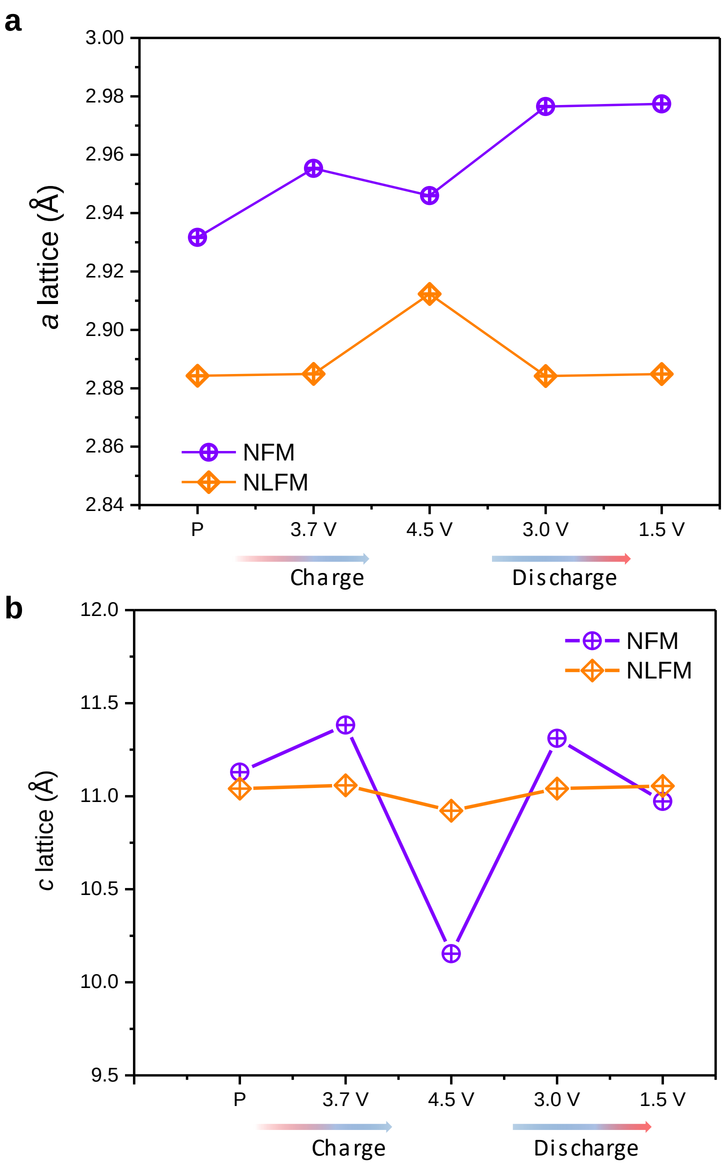

Figure S17. Evolutions of cell parameters of NFM and NLFM during the first cycle. **a.** *a* lattice. **b.** *c* lattice.

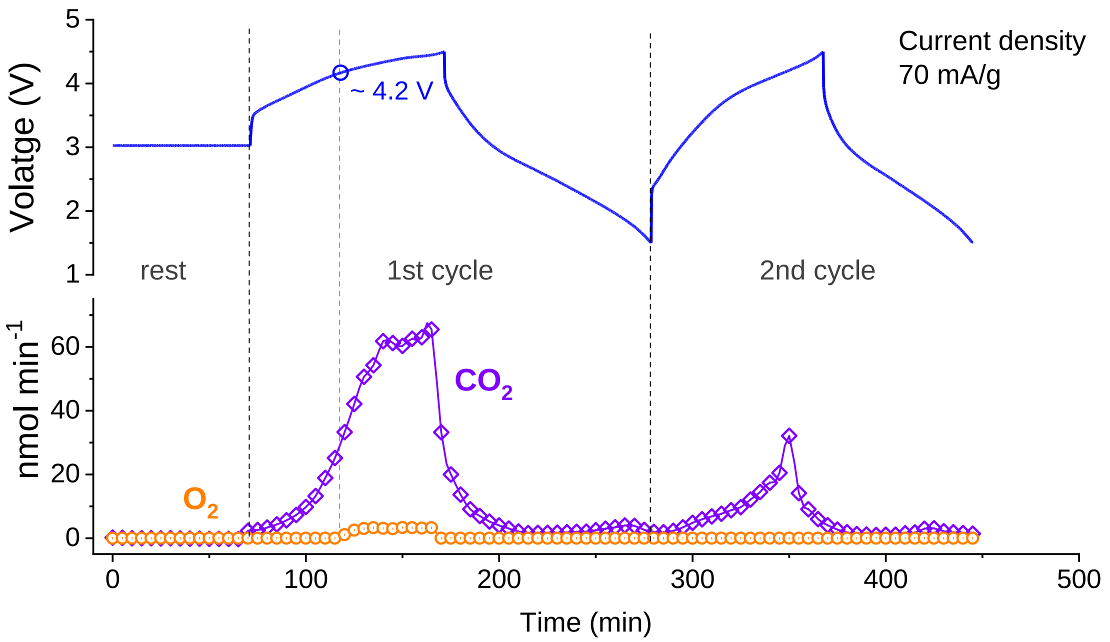

Figure S18. The gas evolution of CO_2 and O_2 for the initial two cycles of NLFM. The electrode was cycled in a galvanostatic model with a current density of 70 mA/g and a potential window of 1.5 - 4.5 V.

Oxygen redox is always accompanied by oxygen loss, which results in irreversible capacity loss. However, the reversible oxygen redox type was also reported. To confirm the oxygen release in NLFM system, the DEMS was performed. When the potential climbs to ~ 4.2 V, there emerges limited oxygen gas. In contrast, CO_2 shows obvious evolution even from the initial charge process. During the second cycle, there was almost no oxygen gas release, while CO_2 displayed the same trend. Combined with the XRD evolution of NLFM, it is confirmed that oxygen release is well suppressed in such a system and has little impact on structure evolution.

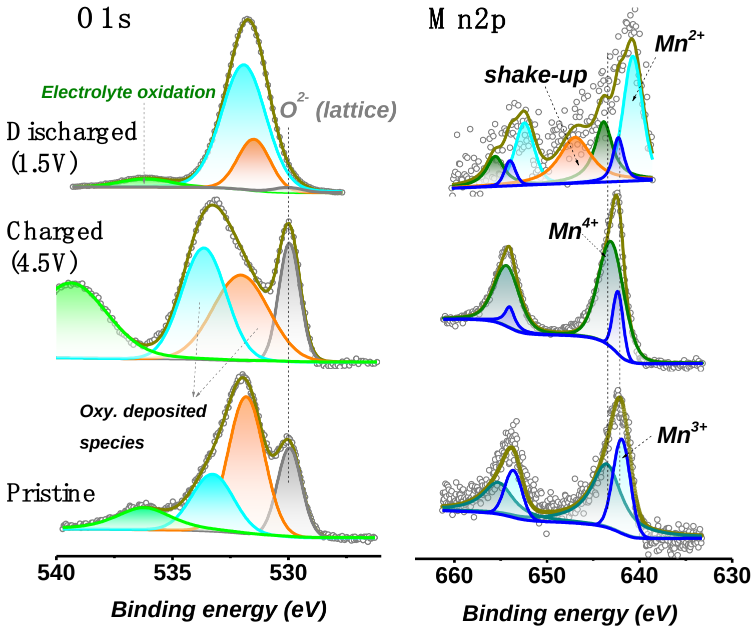

Figure S19. The XPS results of O1s and Mn2p for $Na_{2/3}Fe_{2/3}Mn_{1/3}O_2$ with different electrodes states including pristine, charge to 4.5 V and discharge to 1.5 V, respectively.

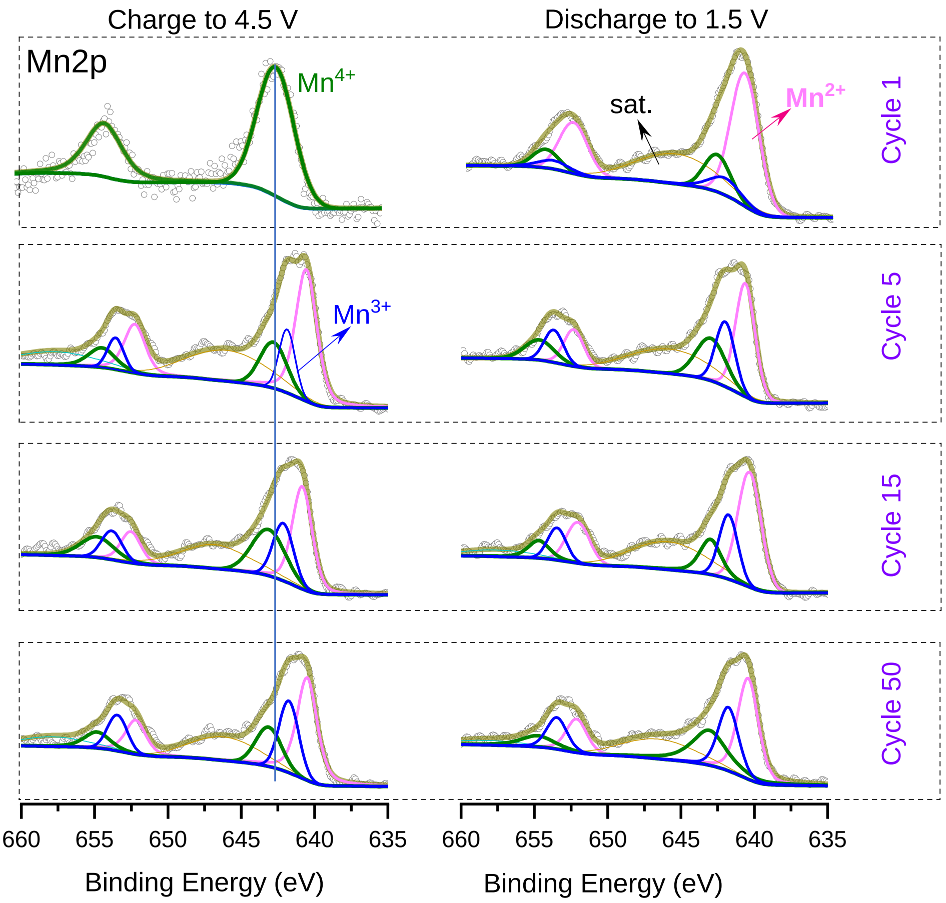

Figure S20. Ex-situ XPS analysis of Mn2p spectra for charged (4.5 V) and discharged (1.5 V) NLFM electrodes over cycling.

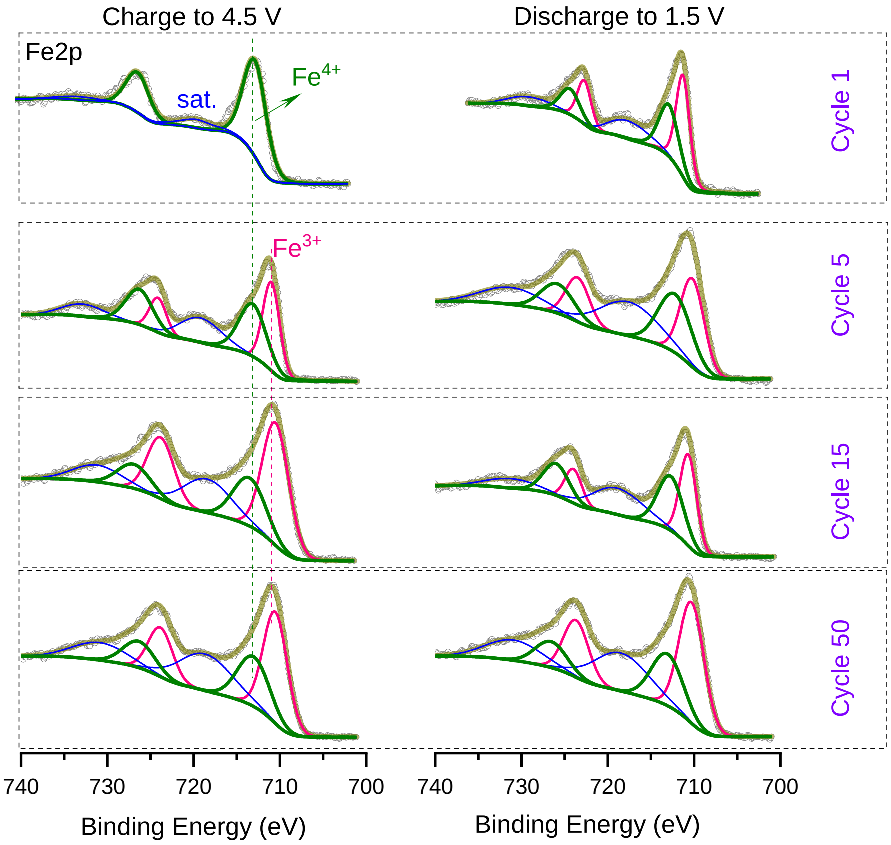

Figure S21. Ex-situ XPS analysis of Fe2p spectra for charged (4.5 V) and discharged (1.5 V) NLFM electrodes over cycling.

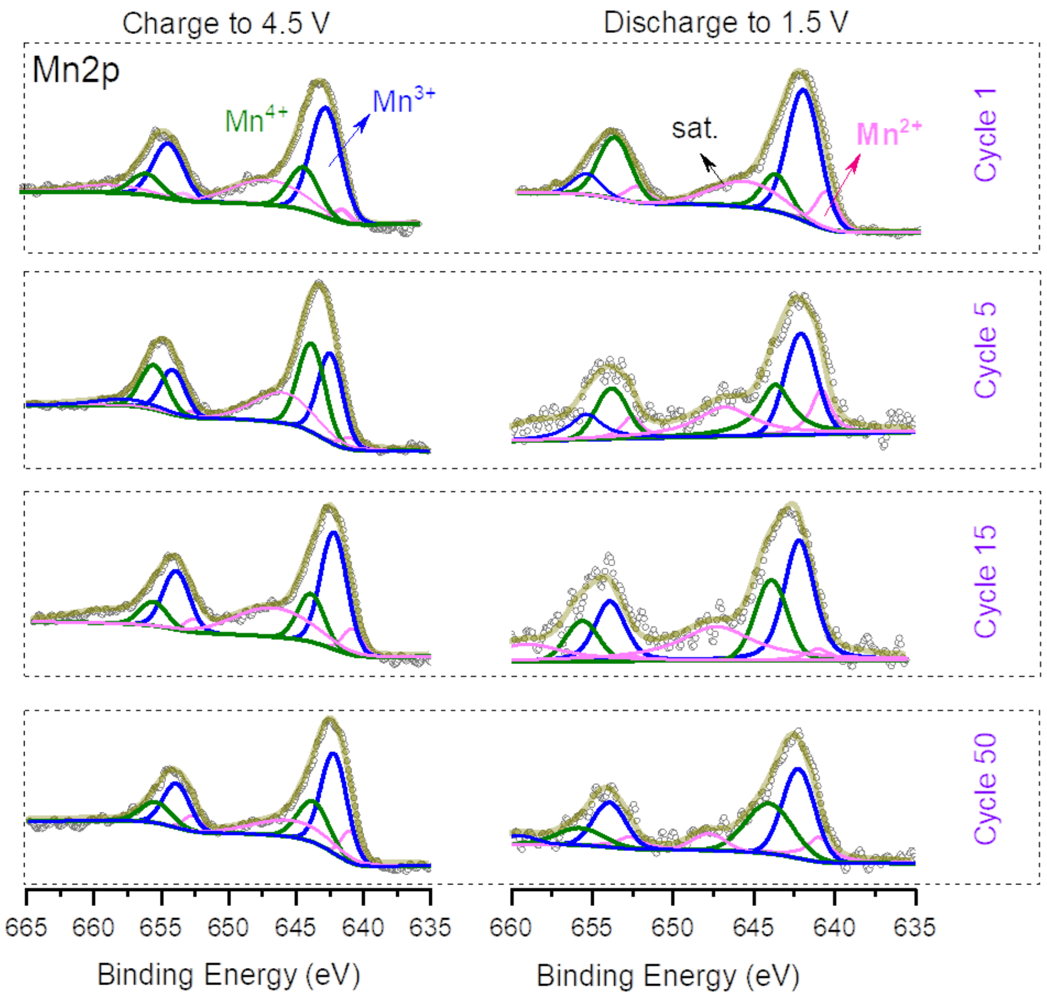

Figure S22. In-depth ex-situ XPS analysis of Mn2p spectra for charged (4.5 V) and discharged (1.5 V) NLFM electrodes over cycling. The electrodes were etched to 300 nm before test.

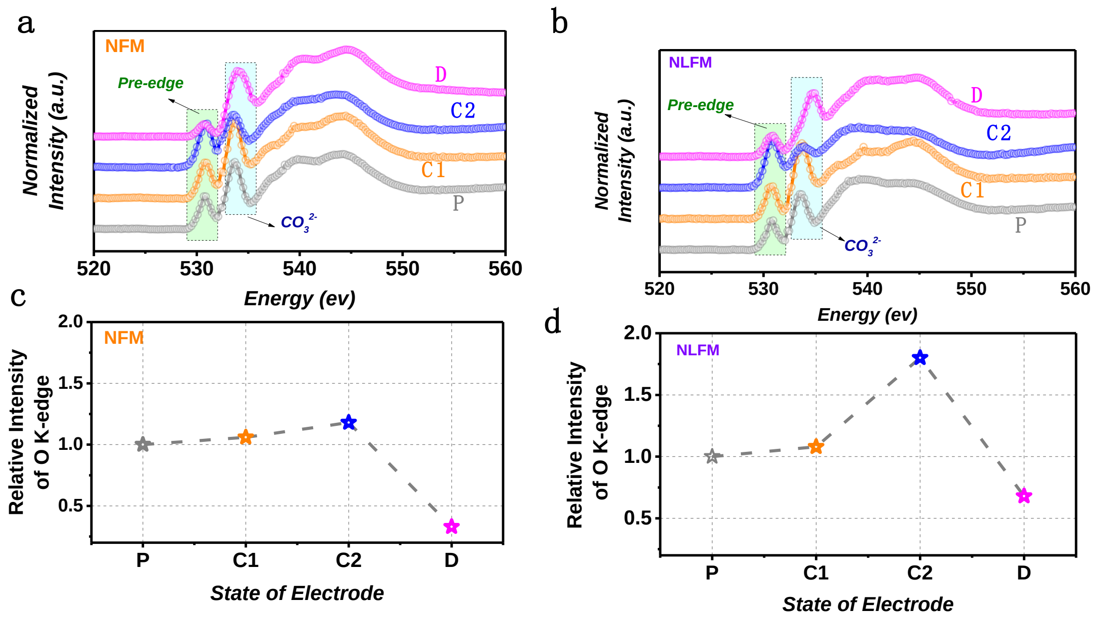

Figure S23. O K-edge sXAS spectra of different states electrodes for **a.** $Na_{2/3}Fe_{2/3}Mn_{1/3}O_2$ (NFM) and **c.** $Na_{0.8}Li_{0.2}Fe_{0.2}Mn_{0.6}O_2$ (NLFM). **b.** The evolution of integrated area for pre-edge peaks for NFM. **d.** The evolution of integrated area for pre-edge peaks for NLFM. The inserted letter P, C1, C2 and D represent the pristine state, the state of charge to 60 mAh g^{-1}, charge to 4.8 V and discharge to 1.5V, respectively.

The pre-edge features before 532 eV are related to the electron transitions from O1s state to hybridized TM 3d-O 2p states. While another section beyond 532 eV is relevant to electron transitions from O1s state to hybridized TM 4sp-O 2p states. The peak locating at ~ 534 eV results from CO_3^{2-} species caused by residual carbonated-salt and/or deposited slat.

References

[1] P. E. Blöchl, O. Jepsen, O. K. Andersen, *Phys. Rev. B* **1994**, *49*, 16223.

[2] G. Kresse, J. Furthmüller, *Physical review. B, Condensed matter* **1996**, *54*, 11169.

[3] G. Kresse, J. Furthmüller, *Comp. Mater. Sci.* **1996**, *6*, 15.

[4] P. E. Blöchl, *Phys. Rev. B* **1994**, *50*, 17953.

[5] V. L. Deringer, A. L. Tchougreeff, R. Dronskowski, *J. Phys. Chem. A* **2011**, *115*, 5461.

[6] H. Allal, Y. Belhocine, E. Zouaoui, *J. Mol. Liq.* **2018**, *265*, 668.

[7] B. He, P. H. Mi, A. J. Ye, S. T. Chi, Y. Jiao, L. W. Zhang, B. W. Pu, Z. Y. Zou, W. Q. Zhang, M. Avdeev, S. Adams, J. T. Zhao, S. Q. Shi, *Acta Mater.* **2021**, *203*, 116490.

[8] S. P. Ong, W. D. Richards, A. Jain, G. Hautier, M. Kocher, S. Cholia, D. Gunter, V. L. Chevrier, K. A. Persson, G. Ceder, *Comp. Mater. Sci.* **2013**, *68*, 314.

[9] A. Y. Toukmaji, J. A. Board, *Comput. Phys. Commun.* **1996**, *95*, 73.

[10] D. H. Lee, J. Xu, Y. S. Meng, *Phys. Chem. Chem. Phys.* **2013**, *15*, 3304.

[11] Y. Hinuma, Y. S. Meng, K. S. Kang, G. Ceder, *Chem. Mater.* **2007**, *19*, 1790.

[12] N. A. Katcho, J. Carrasco, D. Saurel, E. Gonzalo, M. Han, F. Aguesse, T. Rojo, *Adv. Energy Mater.* **2017**, *7*, 1601477.
